# Ecological distribution and population dynamics of Rift Valley fever virus mosquito vectors (Diptera*,* Culicidae) in Senegal

**DOI:** 10.1186/s13071-017-2591-9

**Published:** 2018-01-09

**Authors:** Biram Biteye, Assane G. Fall, Mamadou Ciss, Momar T. Seck, Andrea Apolloni, Moussa Fall, Annelise Tran, Geoffrey Gimonneau

**Affiliations:** 1 Institut Sénégalais de Recherches Agricoles/Laboratoire National de l’Elevage et de Recherches Vétérinaires, BP 2057 Dakar-Hann, Senegal; 2 Université Cheikh Anta Diop, Faculté des Sciences et Techniques, Département de Biologie Animale, Laboratoire d’Ecologie Vectorielle et Parasitaire, Dakar, Senegal; 3 CIRAD, UMR ASTRE, INRA, F-34398 Montpellier, France; 4 CIRAD, UMR TETIS, F-97940 Sainte-Clotilde, Reunion Island France; 5 CIRAD, UMR INTERTRYP, F-34398 Montpellier, France; 6 Centre International de Recherche – Développement sur l’Elevage en zone subhumide, Bobo-Dioulasso 01, BP 454 Burkina Faso

**Keywords:** Ecology, Mosquito vectors, Rift Valley fever virus, Senegal, Biotope, Meteorology

## Abstract

**Background:**

Many zoonotic infectious diseases have emerged and re-emerged over the last two decades. There has been a significant increase in vector-borne diseases due to climate variations that lead to environmental changes favoring the development and adaptation of vectors. This study was carried out to improve knowledge of the ecology of mosquito vectors involved in the transmission of Rift Valley fever virus (RVFV) in Senegal.

**Methods:**

An entomological survey was conducted in three Senegalese agro-systems, Senegal River Delta (SRD), Senegal River Valley (SRV) and Ferlo, during the rainy season (July to November) of 2014 and 2015. Mosquitoes were trapped using CDC light traps set at ten sites for two consecutive nights during each month of the rainy season, for a total of 200 night-traps. Ecological indices were calculated to characterize the different populations of RVFV mosquito vectors. Generalized linear models with mixed effects were used to assess the influence of climatic conditions on the abundance of RVFV mosquito vectors.

**Results:**

A total of 355,408 mosquitoes belonging to 7 genera and 35 species were captured in 200 night-traps. RVFV vectors represented 89.02% of the total, broken down as follows: *Ae. vexans arabiensis* (31.29%), *Cx. poicilipes* (0.6%), *Cx. tritaeniorhynchus* (33.09%) and *Ma. uniformis* (24.04%). Comparison of meteorological indices (rainfall, temperature, relative humidity), abundances and species diversity indicated that there were no significant differences between SRD and SRV (*P* = 0.36) while Ferlo showed significant differences with both (*P* < 0.001). Mosquito collection increased significantly with temperature for *Ae. vexans arabiensis* (*P* < 0.001), *Cx. tritaeniorhynchus* (*P* = 0.04) and *Ma. uniformis* (*P* = 0.01), while *Cx. poicilipes* decreased (*P* = 0.003). Relative humidity was positively and significantly associated with the abundances of *Ae. vexans arabiensis* (*P* < 0.001), *Cx. poicilipes* (*P* = 0.01) and *Cx. tritaeniorhynchus* (*P* = 0.007). Rainfall had a positive and significant effect on the abundances of *Ae. vexans arabiensis* (*P* = 0.005). The type of biotope (temporary ponds, river or lake) around the trap points had a significant effect on the mosquito abundances (*P* < 0.001).

**Conclusions:**

In terms of species diversity, the SRD and SRV ecosystems are similar to each other and different from that of Ferlo. Meteorological indices and the type of biotope (river, lake or temporary pond) have significant effects on the abundance of RVFV mosquito vectors.

**Electronic supplementary material:**

The online version of this article (10.1186/s13071-017-2591-9) contains supplementary material, which is available to authorized users.

## Background

Many human and animal infectious diseases with major impacts on public and veterinary health have emerged or re-emerged over the last two decades [1–3]. Rift Valley fever (RVF) is endemic in many African countries and represents a real threat for European countries. Rift Valley fever virus (RVFV) is an emerging arbovirus considered to be a major public and veterinary health problem. Outbreaks in Africa [4–6] and the Arabian Peninsula [7–9] have had huge economic repercussions in terms of animal deaths and economic losses in the affected countries [10, 11]. RVF is a barrier to economic development in countries where the population is mostly rural and livestock play an important role in their economies. The recent RVF emergence and re-emergence are related to genetic, biological, environmental, climatic, political, economic, demographic and social factors [12–16].

Since the 1987 epidemic in Mauritania, many serological and entomological studies have been conducted in Senegal and the results have highlighted the frequent circulation of RVFV [17–20]. In East Africa, particularly in Kenya, the epidemiological patterns of RVF are different from those described in West Africa, particularly in Senegal. In Kenya, several RVF outbreaks have been linked to prolonged heavy rainfall, whereas in Senegal, outbreaks usually occur during years of normal or poor rainfall [18, 21, 22]. The main East African RVFV vectors are *Ae. ochraceus* and *Aedes mcintoshi* [23–25], while in West Africa the main vectors are *Ae. vexans arabiensis, Ae. ochraceus* and *Cx. poicilipes* [17, 26, 27].

RVF is endemic in Senegal, especially in the North (Ferlo) [19, 28], and the transmission of the virus is seasonal with a peak at the end of the rainy season. This seasonality and the persistence of the virus during inter-epizootic periods may be explained by two possible mechanisms: (i) each year, the virus is introduced into the area at the beginning of the rainy season by transhumant herds coming from neighboring regions to the North and South; (ii) the virus may survive in the area in *Aedes*’ diapausing eggs [29] from the previous rainy season and in overwintering *Culex* populations [30]. RVFV mosquito vectors are numerous and they change depending on the ecosystems involved. In Ferlo ecosystems, *Ae. vexans arabiensis* and *Cx. poicilipes* are the main vectors [18, 27, 31]. *Culex poicilipes* has been considered widely to be the sole major RVFV vector in the Senegal River Valley (SRV) and Senegal River Delta (SRD) [17]. However, Fall et al. [32, 33] have shown that *Cx. poicilipes* is not among the most abundant species in these areas. More specifically, they have shown that *Cx. tritaeniorhynchus* and *Ma. uniformis* are the most abundant species during the rainy season.

This study focused on three sites in northern Senegal representing different ecosystems, namely Ferlo (temporary ponds), SRV and SRD (permanent watercourses). Mosquito population dynamics and composition are strongly affected by the water bodies present in a given area: around temporary ponds, the population dynamics are strongly seasonal, with a peak of abundance occurring when rainfall incidents are separated by several dry days; around rivers and lakes mosquitoes populations are constant over the year and represent an unfavorable environment for *Aedes*. This study aimed to understand the relationship between vector dynamics and climatic and environmental factors by determining the ecological indices of composition and structure of the Culicidae fauna in different ecosystems, comparing the seasonal dynamics of the four most abundant species which are potential vectors of RVFV (*Ae. vexans arabiensis, Cx. poicilipes, Cx. tritaeniorhynchus* and *Ma. uniformis*) and quantifying the effects of climatic and environmental factors.

## Methods

### Study area

**Fig. 1:**
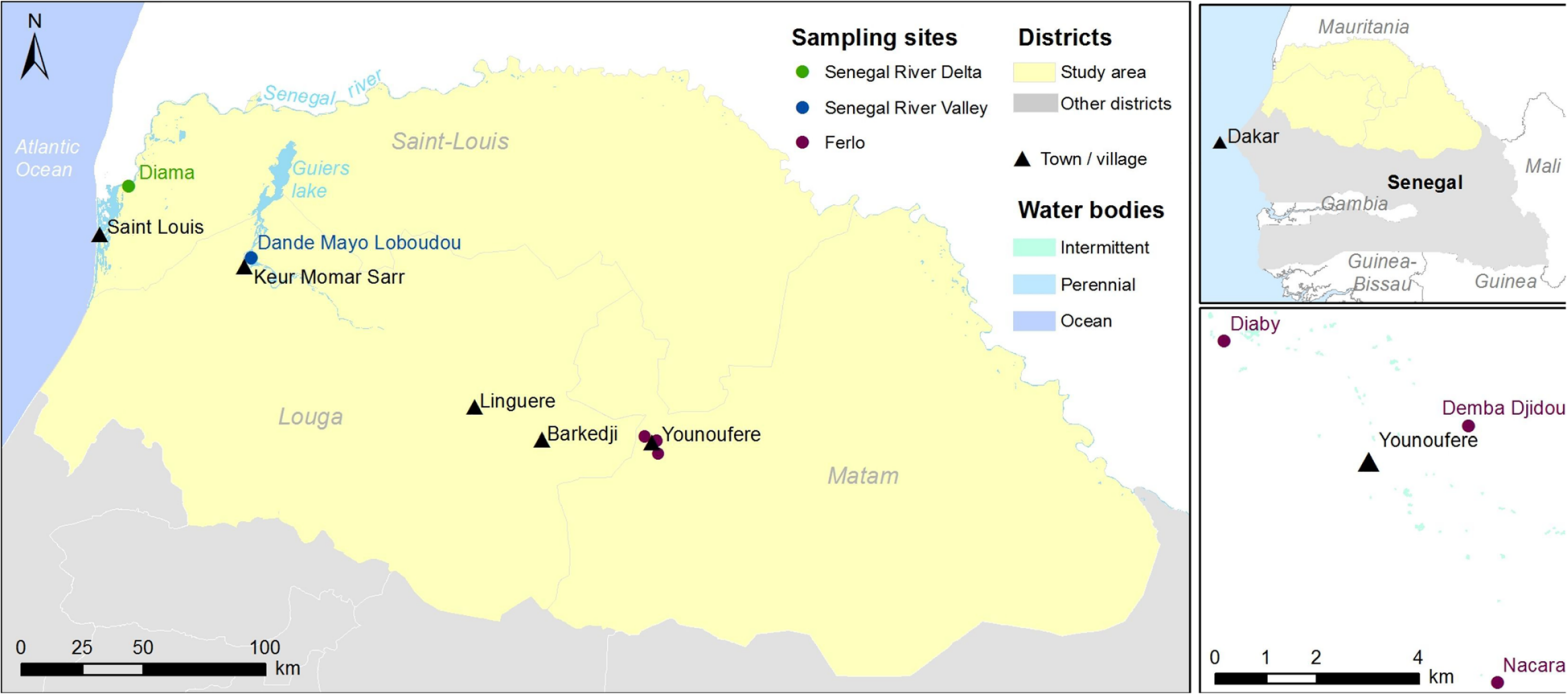
Location of the three sampling sites in northern Senegal. Top-right corner: Senegal map and area of interest (in yellow). Main figure: triangles represent main towns/ villages nearby the sampling sites, while full circles correspond to the sampling points. Bottom-right corner figure: detail of the positions of the three sampling points in Younoufere

Three localities, Diama, Dandé Mayo Loboudou (DML) and Younouféré, were selected in the SRD, SRV and Ferlo ecosystems, respectively (Fig. 1). These sites had all been affected recently by RVF outbreaks [5, 34]. Diama (16°12′41.4′′N, 16°23′31.6′′W), is a small village on the bank of the SRD located 28 km east of the town of St-Louis. The main human activities are agriculture and animal breeding. Traditional farming methods are used and herders practice transhumance. The climate is semi-arid with low rainfall (between 100 and 500 mm/year) during the rainy season (July–October) and a long, nine-month dry season [35]. DML is a village (15°56′51.7"N, 15°56′22.2"W) in SRV located 6 km from Keur Momar Sarr (KMS) town, near Guiers Lake, an important fresh water reserve covering nearly 0.5 km^2^ [36]. The village belongs to a sylvo-pastoral area located immediately south of the river valley and occupying part of the Sahelian and Sudano-Sahelian region. Extensive farming/pasturage is the main production system in the area [36]. Younouféré village (15°16′08.7"N and 14°27′52.5"W) is located in Ferlo. It is surrounded by small hamlets composed of only a few houses. The hamlets of Diaby (15°17′18.1"N, 14°29′07.9"W), Demba Djidou (15°16′53.6"N, 14°27′04.8"W) and Nacara (15°13′23.1"N, 14°26′18.8"W) were selected as the study sites. The area is characterized by a semi-arid steppe and many temporary ponds that are filled by run-off water. These ponds are the main source of water for humans and animals during the rainy season [26, 37], and are also important breeding sites for mosquitoes. The Ferlo region is an important transhumance point for livestock (cattle and small ruminants) coming from Mauritania; the livestock proceed south at the beginning of the rainy season and move north during the dry season.

### Collection of meteorological data

Local veterinarian officers, who routinely collect daily rainfall data using rain gauges located in each location, provided the rainfall data. Temperature and relative humidity were collected on each site every hour of every day throughout the year using a data logger (HOBO U10 Temp / RH Data Logger, West Sussex, United Kingdom).

### Sampling and processing of entomological data

An entomological survey was conducted in 2014 and 2015 during the rainy season (from July to November). Every month, mosquitoes were trapped during two consecutive nights (from 6 PM to 6 AM) in each study site using CO2-baited CDC light traps (BioQuip # 2836Q-6VDC, Rancho Dominguez, USA) placed outdoors_._ This type of trap is used routinely for sampling arbovirus mosquito vectors [18, 31, 37]. Two traps were set per site at a height of about 1.5 m from the ground: one close to a natural water point (river, lake or pond); another close to a livestock pen. The distance between the water source and livestock pen varied from 100 to 800 m depending on the site. In the field, the mosquitoes collected were killed by freezing in dry ice, sorted by genus on a chill table, put in 15 or 50 ml centrifuge tubes/cryo-tubes and transported in dry ice (-80 °C) to the laboratory where they were identified according to sex and species on a chill table (-20 °C) using morphological keys [38, 39] and identification software [40–42].

### Statistical analysis

To characterize the different populations of RVF mosquito vectors, ecological variables were used as predictors. Thus for each site, the following indices were calculated: (i) the ecological indices of composition: total (S) and average (Sm) species richness, total (N) and relative (AR%) abundance, frequency of occurrence or constancy (C%) [8]; and (ii) the ecological indices of structure: Shannon-Weaver diversity index (H′), maximum diversity index (H′ max), Simpson’s diversity index (1-D) and equitability index (E) [43–46]. Since our data were not normally distributed [47], non-parametric Kruskal-Wallis [49], Mann-Whitney-Wilcoxon tests [48, 49], and principal components analysis (PCA) were used to assess differences in species abundances and meteorological variables (temperature, relative humidity and rainfall) between localities and capture points. The Pearson’s correlation test was used to exclude strongly correlated (*r* ≥ 0.9) variables from the analysis. A generalized linear mixed model (GLMM) [50] was used to assess the effect of climatic variables [temperature (mean of capture day), relative humidity (mean of capture day) and rainfall (mean and max. from 6 to 7 days prior to the capture event)] on mosquito abundances; site and date of capture were considered as random effects [51]. The collected data set on the mosquitoes’ temporal abundances was randomly divided into a training set and a test set. The training set was used to build the model and the test set to validate the best model based on the Akaike information criterion (AIC) [52] for each species. A random selection was performed and 2/3 (67%) of the entire data set (see Additional file 1: Table S1) was assigned to the training set and 1/3 (33%) to the test set. All of the analyses were carried out with R software [53], *lme4* package [50] was used to fit the GLMM, *ade4* package [54] to fit the PCA and *vegan* package [55] to fit the ecological indices.

## Results

### Species composition and abundances

A total of 355,408 specimens belonging to seven genera (*Aedes*, *Aedeomyia*, *Anopheles*, *Culex, Culiseta*, *Mansonia* and *Uranotaenia*) and 35 species were captured in 200 night-traps in the three ecosystems (Table 1). RVFV vectors represented 89.02% of the total species captured. The total was broken down as follows: *Cx. tritaeniorhynchus* (33.1%), *Ae. vexans arabiensis* (31.3%), *Ma. uniformis* (24.0%) and *Cx. poicilipes* (0.6%). In the ecosystems of SRD and SRV, characterized by permanent watercourses, *Cx. tritaeniorhynchus* was the most abundant species, accounting for 54.8% of the total capture in SRD and 42.7% in SRV, *Ma. uniformis* accounted for 34.9 and 38.0%, respectively while *Cx. poicilipes* (0.55 and 1.05%, respectively) and *Ae. vexans arabiensis* (0.01 and 0.03%, respectively) were very rare. In the Ferlo ecosystem, *Ae. vexans arabiensis* was the most abundant species (94.98%), while *Cx. tritaeniorhynchus*, *Cx. poicilipes* and *Ma. uniformis* represented 0.29, 0.24 and 0.003%, respectively, of the mosquitoes captured.

**Table 1:** Number of mosquitoes collected for each sampling site in 2014 and 2015

	SRD		SRV		Ferlo						Total	
Species	Diama		DML		Diaby		Djidou		Nacara			
	2014	2015	2014	2015	2014	2015	2014	2015	2014	2015	2014	2015
*Aedes aegypti*	0	1	0	1	9	7	10	6	1	0	20	15
*Ae. argenteopunctatus*	0	0	0	0	0	1	0	3	0	0	0	4
*Ae. circumluteolus*	0	0	0	0	0	0	3	0	0	0	3	0
*Ae. fowleri*	0	0	0	0	1	0	0	15	0	0	1	15
*Ae. mcintoshi*	0	0	0	0	1028	2	63	51	2	8	1093	61
*Ae. minutus*	0	0	0	0	0	0	0	3	0	0	0	3
*Ae. ochraceus*	0	0	0	0	734	92	28	41	9	33	771	166
*Aedes* spp.	0	0	0	0	1	0	16	51	9	1	26	52
*Ae. sudanensis*	0	2	0	2	140	63	52	50	11	10	203	127
*Ae. vexans arabiensis*	0	9	0	30	85,653	3720	15,164	2512	3535	582	104,352	6853
*Aedeomyia africana*	359	458	2	2	1	0	0	0	0	0	343	460
*Anopheles brunnipes*	46	0	0	0	0	0	0	0	0	0	46	0
*An. domicola*	8	0	1860	573	0	0	0	0	0	0	1868	573
*An. flavicosta*	0	0	0	1	0	0	0	0	0	0	0	1
*An. freetownensis*	1	0	0	0	0	0	0	0	0	0	1	0
*An. funestus*	0	3	483	201	1	0	1	0	0	1	485	205
*An. gambiae*	14	16	3	0	8	4	1	6	2	3	28	29
*An. pharoensis*	111	143	249	40	17	16	6	6	3	3	382	208
*An. rufipes*	4	6	19	44	49	68	3	19	0	6	74	143
*Anopheles* spp*.*	6	120	50	5	48	3	1	15	11	7	116	150
*An. squamosus*	3	4	1	0	1136	22	23	28	18	2	1181	56
*An. wellcomei*	2	0	35	50	0	0	0	0	0	0	37	50
*An. ziemanni*	3579	3602	8964	3853	29	82	1	58	0	32	12,213	7764
*Culiseta* spp.	5	0	0	0	0	0	0	0	0	0	5	0
*Culex antennatus*	68	2334	748	915	15	8	0	1	0	1	827	3259
*Cx. bitaeniorhynchus*	0	0	0	0	4	557	22	38	0	30	26	625
*Cx. decens*	46	75	1	1	1	17	4	23	0	0	52	100
*Cx. neavei*	70	6	2152	25	2	0	10	0	9	3	2220	34
*Cx. perfuscus*	362	439	0	0	0	0	0	0	0	0	358	439
*Cx. poicilipes*	120	581	791	386	18	50	46	1	115	56	1065	1074
*Cx. quinquefasciatus*	43	31	254	27	0	4	3	2	0	0	295	64
*Cx. sitiens*	304	39	1	0	0	0	0	0	0	0	305	39
*Culex* spp*.*	5	2	24	0	39	4	2	22	3	7	73	35
*Cx. theileri*	0	1	0	0	0	3	0	0	0	0	0	4
*Cx. tritaeniorhynchus*	29,400	40,080	37,580	10,341	165	13	33	54	31	24	67,085	50,512
*Cx. univittatus*	1	3	6	9	43	14	0	8	0	2	50	36
*Cx. ventrilloni*	4	0	0	0	0	0	0	0	1	0	5	0
*Mansonia uniformis*	16,495	27,754	28,299	14,302	2	2	2	0	0	0	43,392	42,053
*Uranotaenia spp*	1	1	0	0	2	0	0	0	0	0	3	1
*Ur. unguculata*	1	0	0	0	0	0	0	0	0	0	1	0
Subtotal	51,058	75,707	81,514	30,808	89,144	4750	15,494	2997	3760	813	240,333	115,075
Total	126,119		112,330		93,894		18,491		4573		355,408	

### Mosquito species diversity and richness

The ecosystem of SRD presented the highest species diversity with S = 27 species collected during the two years (average Sm = 9.7 ± 0.21 per species and H′ max = 4.43 ± 0.05 bits), followed by the Ferlo ecosystem (S = 26 species, Sm = 3.7 ± 0.2 species and H′ max = 4.52 bits), and SRV (S = 22 species, Sm = 8.5 ± 0.2 species and H′ max = 4.21 ± 0.05 bits). However, according to the diversity index (H′), SRV was the most diversified ecosystem (H′ = 1.318 bit for 2014 and 1.33 bit for 2015) followed by SRD (H′ = 1.037 and 1.087 bit, respectively) and Ferlo (H′ = 0.254 and 0.332 bit, respectively). This trend was verified by the Simpson’s diversity index (Table 2). Independently of the year and the ecosystem, the values of equitability (E) approached zero, reflecting an unbalanced population dominated by only one species: *Cx. tritaeniorhynchus* in SRD and SRV and *Ae. vexans arabiensis* in the Ferlo area. Comparing the ecological indices of composition and structure (Table 2) for Diama (SRD) and DML (SRV), we found that there was no significant difference (W = 895.5, *P* = 0.36) in abundances and species diversity for the two sites. However, mosquito abundances and diversity in the Ferlo area were significantly different from those of SRD (W = 4453, *P* < 0.001) and SRV (W = 4299, *P* < 0.001). The same was observed for the abundances of RVF mosquito vectors (*Ae. vexans arabiensis*, *Cx. poicilipes*, *Cx. tritaeniorhynchus* and *Ma. uniformis*) in the three ecosystems. These test results were supported by a principal components analysis (PCA) whose first four axes contained 80.24% of the total inertia. With a permutation test (*P* < 0.001), the variance of the interclass analysis (between sites) explains 11.58% of PCA variance, against 88.42% for intraclass analysis (between trap points or biotopes), showing that the effect of the biotope was more important on mosquito abundances and diversity than the effect of the site.

**Table 2:** Ecological indices of composition and structure by study site in 2014–2015

Locality		Diama (SRD)		DML (SRV)		Younouféré (Ferlo)	
Year		2014	2015	2014	2015	2014	2015
Abundance (N)		51,058	75,707	81,514	30,808	108,475	8551
Total richness (S)		22	21	18	19	23	23
Average richness (Sm)		9.85	9.55	8.6	8.35	3.57	3.82
Maximum diversity (H′ max)		4.459	4.392	4.169	4.247	4.523	4.523
Shannon index (H′)		1.037	1.087	1.318	1.33	0.254	0.332
Simpson’s index (1-D)		0.446	0.427	0.346	0.34	0.921	0.898
Equitability index (E)		0.233	0.247	0.316	0.313	0.056	0.073
AR (%)	*Ae. vexans arabiensis*	0	0.012	0	0.097	96.19	79.04
	*Cx. poicilipes*	0.235	0.767	0.974	1.252	0.165	1.251
	*Cx. tritaeniorhynchus*	57.581	52.94	46.1	33.57	0.228	1.064
	*Ma. uniformis*	32.306	36.659	34.71	46.42	0.003	0.02
C (%)	*Ae. vexans arabiensis*	0	20	0	25	68.33	61.66
	*Cx. poicilipes*	75	65	90	70	16.66	15
	*Cx. tritaeniorhynchus*	100	100	100	95	56.66	25
	*Ma. uniformis*	100	100	100	95	6.666	1.66

*Abbreviations*: *AR* relative abundance, *C* frequency of occurrence or constancy

### Seasonal dynamics of RVFV mosquito vectors

**Fig. 2:**
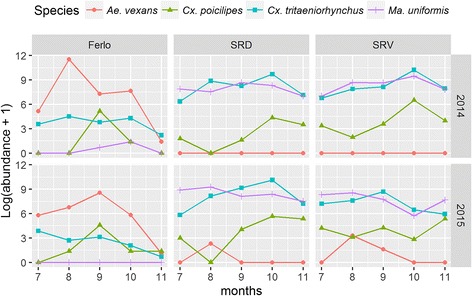
Seasonal population dynamics of RVF mosquito vectors in northern Senegal per year (row) and site (column). On the x-axis we report the time (months) of mosquitoes’ capture; on the y-axis the corresponding abundance (logarithmic scale); each color correspond to a vector species. *Abbreviations*: SRD, Senegal River Delta; SRV, Senegal River Valley

Mosquito dynamics showed significant seasonal differences. In fact, non-parametric Kruskal-Wallis and Mann-Whitney-Wilcoxon tests have shown that the abundances of mosquitoes changed significantly over the study period (*χ*^2^ = 32.41, *df* = 4, *P* < 0.001) and between ecosystems (*χ*^2^ = 97.77, *df* = 2, *P* < 0.001). Depending on the year, a significant difference was observed in mosquito abundances; there were more mosquitoes in 2014 than in 2015 (Table 1) although there was more rainfall in 2015 (258.4, 301.9 and 292.3 mm in SRD, SRV and Ferlo, respectively) than in 2014 (70.4, 147.1 and 246.2 mm, respectively). Thus while *Aedes* species, in particular *Ae. vexans arabiensis,* were only present in Ferlo in 2014, they were present in all three ecosystems during the 2015 rainy season (Fig. 2, Table 1). *Culex poicilipes*, *Cx. tritaeniorhynchus* and *Ma. uniformis* were present in the three ecosystems throughout the two years, albeit with very different abundances (Fig. 2, Table 2). *Aedes vexans arabiensis* appeared at the beginning of the rainy season and reached peaks of abundance in August (2014) and September (2015) in the three ecosystems (Fig. 2) but abundances decreased considerably moving North. *Culex poicilipes* populations appeared during the second half of the rainy season and reached a peak in September in the Ferlo area and in October in SRD and SRV (Fig. 2). Unlike *Ae. vexans arabiensis*, *Cx. poicilipes* abundances increased considerably moving North. On the other hand, the population abundance of *Ma. uniformis* and *Cx. tritaeniorhynchus* remained unchanged during the rainy season (Fig. 2).

### Effects of climate variables on the abundance of RVFV mosquito vectors

Results from the GLMM (Table 3) showed that temperature (mean of capture day), relative humidity (mean of capture day) and rainfall (mean from 6 to 7 days prior the capture event) were significantly related to the abundances of *Ae. vexans arabiensis* populations (*P* ≤ 0.005). The daily average of relative humidity increased abundances of *Cx. poicilipes* populations (*P* = 0.017) while the temperature decreased abundances (*P* = 0.003). Temperature had a positive and significant effect on the abundance of *Cx. tritaeniorhynchus* (*P* = 0.048) and *Ma. uniformis* populations (*P* = 0.014). An increase in the abundance of *Ma. uniformis* was observed when there was heavy rain (max. rains from 6 to 7 days prior the capture event) but the effect was not significant (*P* = 0.384). The daily average of relative humidity increased the abundance of *Cx. tritaeniorhynchus* (*P* = 0.007) and decreased those of *Ma. uniformis* even if the effect was not significant (*P* = 0.588). The type of biotope (river, lake or ponds) has significant effect on the mosquito abundances (*P* < 0.001) and explains 88.42% of PCA variance.

**Table 3:** Poisson-GLMM abundance model used for each of the four potential RVFV vectors

	Regression coefficient	SE	*Z*-value	*P*-value
*Ae. vexans arabiensis*				
Intercept	-23.08933	4.8033	-4.807	1.53e-06
Temperature (mean of capture day)	0.59791	0.15218	3.929	8.53e-05
Humidity (mean of capture day)	0.08697	0.02454	3.543	0.000395
Rainfall (mean from 6 to 7 days prior to the capture event)	0.07836	0.02837	2.762	0.005737
*Cx. poicilipes*				
Intercept	11.06897	5.24993	2.108	0.035
Temperature (mean of capture day)	-0.54716	0.18934	-2.89	0.00385
Humidity (mean of capture day)	0.06626	0.02797	2.369	0.01783
*Cx. tritaeniorhynchus*				
Intercept	-5.88329	3.27343	-1.797	0.07229
Temperature (mean of capture day)	0.20359	0.10296	1.977	0.04800
Humidity (mean of capture day)	0.04447	0.01672	2.659	0.00783
*Ma. uniformis*				
Intercept	-9.380981	5.716084	-1.641	0.1008
Temperature (mean of capture day)	0.242669	0.099457	2.44	0.0147
Humidity (mean of capture day)	-0.010763	0.019884	-0.541	0.5883
Rainfall (max from 6 to 7 days prior to the capture event)	0.005822	0.00669	0.87	0.3842

*Abbreviation*: *SE* standard error

## Discussion

We studied the dynamics of Culicidae mosquito communities in three different ecosystems during the same period for two consecutive years, using ecological indices of composition and structure, comparative dynamics, PCA and linear regression models. To the best of our knowledge, this is the first time that such an approach has been used in West Africa to compare ecological dynamics of the same species of mosquitoes in different ecosystems which have recently experienced RVF outbreaks [5, 34]. *Aedes vexans arabiensis* and *Cx. poicilipes* have been identified as the main RVFV vectors in Ferlo [17, 31, 56]; *Cx. tritaeniorhynchus* and *Ma. uniformis* are highly suspected to be involved in RVFV transmission in SRD and SRV because of their abundance [32, 33] and frequent infections with RVFV [9, 37].

Our study shows that *Cx. tritaeniorhynchus* and *Ma. uniformis* were the most abundant and dominant species in SRD and SRV. These observations are similar to those of Fall et al. [32, 33] in Ross Béthio (SRD) and those of Diallo et al. [56] along the SRV for *Ma. uniformis*. *Culex poicilipes* was poorly represented in our study (0.55% for SRD and 1.05% for SRV), while in Diallo et al. [56] this species was the most represented (41%). These observations could be explained by the fact that *Cx. tritaeniorhynchus* and *Ma. uniformis* breed generally in fresh and permanent waters colonized by aquatic plants, while *Cx. poicilipes* prefers less contaminated waters. Our results show more species than Fall et al. [32] in SRD (28 species vs 12 species) and less species than Diallo et al. [56] in SRV (22 species vs 41 species). In relation to Fall et al. [32], the difference may be attributed to the type of trap used: the previous studies used animal-baited traps that are host-specific trapping methods [32, 57], while we used CDC traps that are more generic and collect the majority of the mosquito species in a given area. In relation to the study by Diallo et al. [56], an entomological surveillance project in SRV, the three field works involved covered a larger area than our study.

With regard to Culicidae diversity, we identified 25 mosquito species in Ferlo, similar to that found by previous studies in the area. In terms of abundance, *Ae. vexans arabiensis* represented 95% of the catches and *Cx. poicilipes* only 0.24%. These observations are in contrast with those of Talla et al. [58] who found that *Cx. poicilipes* was the most abundant species. It is known that rainfall affects the abundance of this species [59]. We can assume that changes in the rainfall and temperature conditions between the two study periods could have impacted the abundance. Differences in the physico-chemical characteristics of the type of breeding sites also might explain this difference [31].

The ecological indices of composition (S, Sm, N, AR % and C %) and the principal components analysis show that the ecosystems of SRD and SRV have similar entomological and meteorological characteristics; they differ from the Ferlo ecosystem in terms of abundance and species diversity. In the SRD and SRV ecosystems, mosquito communities are mainly dominated by *Cx. tritaeniorhynchus* and *Ma. uniformis* while in the Ferlo ecosystem, *Ae. vexans arabeinsis* is dominant. The same ecological indices showed significant differences in mosquito abundance and diversity across the three zones, suggesting that these ecological zones are potential risk areas for RVF transmission and circulation. The same conclusion has been drawn by Arum et al. [25] on the abundance of RVF mosquito vectors along livestock movement routes in the northeastern and coastal regions of Kenya.

The ecological indices of structure, 1-D and the most commonly used H′ [60], together with E index, show that the three ecosystem conditions are adverse to the development of mosquito communities. The low level of H′ (< 3) gives an indication of the ecological state of the environment. According to Simboura & Zenetos [61], the three ecosystems are all heavily polluted. This may further explain the low abundances of *Cx. poicilipes* whose larvae usually develop in sites full of erect vegetation where water is soft and little polluted. According to our observations, independent of the year and ecosystem, the values of equitability index (E) approached zero [62], thus reflecting an unbalanced population, dominated by only one species [44, 45]: *Cx. tritaeniorhynchus* in SRD and SRV and *Ae. vexans arabiensis* in Ferlo. This imbalance can be related to the fluctuations of the climatic parameters, e.g. rainfall directly affecting mosquitoes’ biology.

In SRD and SRV, two ecosystems with permanent watercourses, a scarcity of *Aedes* mosquitoes limits their role in RVF epidemiology. In these areas, Culicidae fauna are dominated by *Culex*, whose population persists throughout the year, and the transmission of RVFV could be continuous with peaks corresponding to high abundances of *Culex* vectors. SRV and SRD are grazing areas. During transhumance, susceptible and infectious individuals (humans and animals) can gather in these two areas. Combined with the high abundance of mosquitoes, increased transmission can trigger local outbreaks. Moreover, the possible transovarial transmission described in *Aedes* [29] and *Culex* mosquitoes [30] could explain the maintenance of RVFV in the two areas. The ecology of RVFV in these ecosystems depends on both the bioecology of the vector and the characteristics of the hosts (their susceptibility to RVF, their mobility and their adaptability to environmental conditions). Due to their abundances, their populations’ stability during the rainy season (population size slowly changes during the season) and their association with RVFV [9, 37], *Cx. tritaeniorhynchus* and *Ma. uniformis* can be considered as potential RVFV vectors in SRD and SRV during the rainy season. This is in contrast to the conclusions by Diallo et al. [17] who identified *Cx. poicilipes* as the main RVF vector in SRV.

Population dynamics of the mosquitoes in the three study areas were significantly different. In pond systems of Ferlo, *Ae. vexans arabiensis* appeared at the first rains and reached the peak of abundance around the middle of the rainy season (August-September), while *Culex,* in particular *Cx. poicilipes,* made their appearance and reached their maximum in September-October. In the permanent watercourse systems of SRD and SRV, *Cx. tritaeniorhynchus*, *Cx. poicilipes* and *Ma. uniformis* reached their peak of abundance at the end of the rainy season. These observations are in agreement with those of Diallo [63] and Mondet et al. [59, 64] in Barkedji, and those of Fall et al. [32] in Ross Béthio.

The influence of climatic conditions on the bioecology of arbovirus vectors has been previously documented [65–67]. We highlighted the complex relationship between rainfall and abundance for *Ae. vexans arabiensis*. Many studies have suggested that the first wintering rainfalls and those immediately after long rainless periods have a positive influence on *Ae. vexans arabiensis*’ abundance [65, 68]. However, more recent studies suggested that the abundance of *Ae. vexans arabiensis* is not only influenced by rainfall [31]. The mean temperature and the relative humidity have a direct and positive effect on the biology of *Ae. vexans arabiensis*. This is further supported by laboratory studies which confirm the influence of varying temperatures on the development of *Ae. albopictus* [69]. On the other hand, the daily mean temperature has a negative effect on the abundance of *Cx. poicilipes* and remains the most influential factor on the biology of this species, as observed in other studies [16]. The current study shows that the dynamics of *Cx. tritaeniorhynchus* and *Ma. uniformis* were positively associated with temperature (both species) and humidity (for *Cx. tritaeniorhynchus*) which concurs with Fall et al. [32]. They showed that *Cx. tritaeniorhynchus* dynamics were correlated with temperature and relative humidity, and the vector density reached the minimum when humidity and temperature were below 55% and 20 °C, respectively.

Our study presents some limitations that we plan to overcome with future fieldwork. One of the limitations is the study’s timeline which was focused on the rainy season. Yet some mosquito species (and potential RVFV vectors) reach their peak of abundance after the rainy season (such as *Cx. poicilipes*). This study did not consider the effects of anthropogenic factors on mosquito abundance that are known to play a major role in the spread of RVFV. Future studies should collect information about land and water use, husbandry practices, livestock movements and landscape changes [using satellite-derived environmental indices Normalized Difference Vegetation Index (NDVI) and Normalized Difference Water Index (NDWI)] in the surroundings of the trap and use this information as predictors for mosquito abundance. Finally, this study focused only on the entomological/ecological component of RVFV transmission, identifying potential RVFV vectors in the ecosystems. To strengthen the conclusions, future entomological work should be coupled with vector competence studies and serological/ virological analyses of sentinel herds in the traps’ surroundings.

## Conclusions

This study contributes to existing knowledge regarding the relationship between RVF vector dynamics and drivers in northern Senegal. This information is critical when planning surveillance and prevention activities in Senegal, and in many African countries, where resources are limited. In terms of abundance and species diversity, there are no significant differences between SRD and SRV, while Ferlo shows significant differences with the other two ecosystems. Environmental and climatic factors significantly affect the abundance of RVF mosquito vectors.

## Additional file

Additional file 1: Table S1. Dates of collections, names of sampling sites, geographical coordinates, abundance of mosquito species collected/date, and environmental variables for mosquito collections at the three sampling sites in Senegal. (XLSX 38 kb)

## References

[1] Jones KE, Patel NG, Levy MA, Storeygard A, Balk D, Gittleman JL (2008). Global trends in emerging infectious diseases. Nature.

[2] Morens DM, Folkers GK, Fauci AS (2004). The challenge of emerging and re-emerging infectious diseases. Nature.

[3] Patz JA, Graczyk TK, Geller N, Vittor AY (2000). Effects of environmental change on emerging parasitic diseases. Int J Parasitol.

[4] Nabeth P, Kane Y, Abdalahi MO, Diallo M, Ndiaye K, Ba K (2001). Rift Valley fever outbreak, Mauritania, 1998: seroepidemiologic, virologic, entomologic, and zoologic investigations. Emerg Infect Dis..

[5] OIE. Rift Valley fever, Dorcas gazelle - Senegal: (SL) 1st report, OIE 20130924. 2013. http://www.promedmail.org. Accessed on 30 Mar 2015.

[6] Woods CW, Karpati AM, Grein T, McCarthy N, Gaturuku P, Muchiri E (2002). An outbreak of Rift Valley fever in northeastern Kenya, 1997–98. Emerg Infect Dis..

[7] MMWR (2000). Outbreak of Rift Valley Fever - Saudi Arabia, August-October, 2000. Ctr Dis Control Prev.

[8] Nanyingi MO, Munyua P, Kiama SG, Muchemi GM, Thumbi SM, Bitek AO (2015). A systematic review of Rift Valley fever epidemiology 1931–2014. Infect Ecol Epidemiol.

[9] Jupp PG, Kemp A, Grobbelaar A, Leman P, Burt FJ, Alahmed AM (2002). The 2000 epidemic of Rift Valley fever in Saudi Arabia: mosquito vector studies. Med Vet Entomol.

[10] Jouan A, Le Guenno B, Digoutte JP, Philippe B, Riou O, Adam F (1988). An RVF epidemic in southern Mauritania. Ann Inst Pasteur Virol.

[11] Peyre M, Chevalier V, Abdo-Salem S, Velthuis A, Antoine-Moussiaux N, Thiry E, et al. A systematic scoping study of the socio-economic impact of Rift Valley fever: research gaps and needs. Zoonoses Public Health. 2013;62(5):309-25. 10.1111/zph.12153 25256804

[12] Bouloy M, Weber F (2010). Molecular biology of rift valley fever virus. Open Virol J.

[13] Clements AC, Pfeiffer DU, Martin V, Pittiglio C, Best N, Thiongane Y (2007). Spatial risk assessment of Rift Valley fever in Senegal. Vector-Borne Zoonotic Dis.

[14] Patz JA, Daszak P, Tabor GM, Aguirre AA, Pearl M, Epstein J, et al. Unhealthy landscapes: policy recommendations on land use change and infectious disease emergence. Environ Health Perspect. 2004;112(10):1092–8. 10.1289/ehp.6877 PMC1247383 15238283

[15] Sang R, Lutomiah J, Said M, Makio A, Koka H, Koskei E, et al. Effects of irrigation and rainfall on the population dynamics of Rift Valley fever and other arbovirus mosquito vectors in the epidemic-prone Tana River County, Kenya. J Med Entomol. 2017;54(2):460-70. 10.1093/jme/tjw206 PMC5850818 28011732

[16] Talla C, Diallo D, Dia I, Ba Y, Ndione J-A, Morse AP (2016). Modelling hotspots of the two dominant Rift Valley fever vectors (*Aedes vexans* and *Culex poicilipes*) in Barkédji, Sénégal. Parasit Vectors.

[17] Diallo M, Lochouarn L, Ba K, Sall AA, Mondo M, Girault L (2000). First isolation of the Rift Valley fever virus from *Culex poicilipes* (Diptera: Culicidae) in nature. Am J Trop Med Hyg.

[18] Fontenille D, Traore-Lamizana M, Diallo M, Thonnon J, Digoutte JP, Zeller HG (1998). New vectors of Rift Valley fever in West Africa. Emerg Infect Dis..

[19] Chevalier V, Lancelot R, Thiongane Y, Sall B, Diaité A, Mondet B (2005). Rift Valley fever in small ruminants, Senegal, 2003. Emerg Infect Dis..

[20] Thonnon J, Picquet M, Thiongane Y, Lo M, Sylla R, Vercruysse J (1999). Rift valley fever surveillance in the lower Senegal river basin: update 10 years after the epidemic. Tropical Med Int Health.

[21] ANACIM. “Nawette” - Veille Agroclimatique Mensuelle du Sénégal. Agence Nationale de l’Aviation Civile et de la Météorologie (ANACIM), Aéroport Léopold Sédar SENGHOR, Dakar-Yoff. issue N°4, Septembre 2013, 6 pp.

[22] Ndione J-A, Bicout DJ, Mondet B, Lancelot R, Sabatier P, Lacaux J-P (2005). Conditions environnementales associées à l’émergence de la fièvre de la vallée du Rift (FVR) dans le delta du fleuve Sénégal en 1987. Environ Risque Sante.

[23] Linthicum K, Davies F, Bailbey C, Kairo A (1983). Mosquito species succession in a dambo in an East African forest. Mosq News.

[24] Linthicum K, Davies F, Bailey C, Kairo A (1984). Mosquito species encountered in a flooded grassland dambo in Kenya. Mosq News.

[25] Arum SO, Weldon CW, Orindi B, Landmann T, Tchouassi DP, Affognon HD (2015). Distribution and diversity of the vectors of Rift Valley fever along the livestock movement routes in the northeastern and coastal regions of Kenya. Parasit Vectors.

[26] Diallo D, Ba Y, Dia I, Konaté L, Diallo M (2008). Utilisation de boeufs traités aux insecticides dans la lutte contre les vecteurs des virus de la fièvre de la vallée du Rift et de la fièvre West Nile au Sénégal. Bull Soc Pathol Exot.

[27] Sow A, Faye O, Ba Y, Diallo D, Fall G, Faye O, et al. Widespread Rift Valley fever emergence in Senegal in 2013–2014. Open Forum Infect Dis. 2016;3(3):ofw149. 10.1093/ofid/ofw149 PMC5047427 27704007

[28] Chevalier V, Thiongane Y, Lancelot R (2009). Endemic transmission of Rift Valley fever in Senegal. Transbound Emerg Dis.

[29] Linthicum KJ, Davies FG, Kairo A, Bailey CL. Rift Valley fever virus (family *Bunyaviridae*, genus *Phlebovirus*). Isolations from Diptera collected during an inter-epidemic period in Kenya. J Hyg (Lond). 1985;95(1):197–209. 10.1017/s0022172400062434 PMC2129511 2862206

[30] Seufi AM, Galal FH (2010). Role of *Culex* and *Anopheles* mosquito species as potential vectors of rift valley fever virus in Sudan outbreak, 2007. BMC Infect Dis.

[31] Diallo D, Talla C, Ba Y, Dia I, Sall AA, Diallo M (2011). Temporal distribution and spatial pattern of abundance of the Rift Valley fever and West Nile fever vectors in Barkedji, Senegal. J Vector Ecol..

[32] Fall AG, Diaïté A, Lancelot R, Tran A, Soti V, Etter E (2011). Feeding behaviour of potential vectors of West Nile virus in Senegal. Parasit Vectors.

[33] Fall AG, Diaïté A, Seck MT, Bouyer J, Lefrançois T, Vachiéry N (2013). West Nile virus transmission in sentinel chickens and potential mosquito vectors, Senegal river delta, 2008–2009. Int J Environ Res Public Health.

[34] OIE. Rift Valley fever - Senegal: (DK) bovine. 2014. http://www.promedmail.org. Accessed on 30 Mar 2015.

[35] Thiongane Y, Thonnon J, Zeller H, Lo MM, Faty A, Diagne F, et al. Données récentes de l'épidémiologie de la Fièvre de la Vallée du Rift (FVR) au Sénégal. Dakar Médical: Bulletin de la Société Médicale d'Afrique Noire de Langue Française, (Spécial Quarantenaire 1996), 1-6. 14520977

[36] CSE. Caracterisation des systèmes de production agricole au Sénégal:Document de synthèse. Dakar: Centre de Suivi Ecologique; 2007. p. 38.

[37] Ba Y, Sall A, Diallo D, Mondo M, Girault L, Dia I (2012). Re-emergence of Rift Valley fever virus in Barkedji (Senegal, West Africa) in 2002–2003: identification of new vectors and epidemiological implications. J Am Mosq Control Assoc.

[38] Diagne N, Fontenille D, Konate L, Faye O, Lamizana M, Legros F (1994). Les anophèles du Sénégal: liste commentée et illustrée. Bull Soc Pathol Exot.

[39] Edwards FW (1941). Mosquitoes of the Ethiopian Region. III. - Culicine adults and pupae.

[40] Brunhers J, Rhaim A, Geoffroy B, Angel G, Hervy JP. Les culicidae de l’Afrique Méditerranéenne: Un programme d’identification et d’enseignement. IRD éditions, vol. IRD; 1999.

[41] Hervy JP, Le Goff G, Geoffroy B, Hervé JP, Manga L, Brunhers J (1998). Les Anophèles de la région afro-tropicale: un logiciel d’identification et d’enseignement.

[42] Schaffner F, Angel G, Geoffroy B, Hervy JP, Rhaiem A, Brunhers J: Les moutiques d’Europe: Programme d’enseignement et d’identification. In: IRD éditions, vol. IRD; 2001.

[43] Dajoz R (1985). Précis d’Écologie.

[44] Faurie C, Ferra C, Medori P, Devaux J (2003). Écologie-approche scientifique et pratique.

[45] Ramade F (2003). Eléments d’écologie - ecologie fondamentale.

[46] Gardener M. Community ecology: analytical methods using R and Excel: Pelagic Publishing Ltd. England (South West); 2014.

[47] Cornillon PA, Guyader A, Husson F, Jégou N, Josse J, Kloareg M, et al. Statistiques avec R. 2e édition augmentée. France; 2010. p. 263. ISBN 978–2–7535–1087–6.

[48] Bauer DF (1972). Constructing confidence sets using rank statistics. J Am Stat Assoc.

[49] Hollander M, Wolfe DA. Nonparametric statistical methods. New York: John Wiley & Sons; 1973.

[50] Bates D, Mächler M, Bolker B, Walker S (2015). Fitting linear mixed-effects models using lme4. J Stat Softw.

[51] Diarra M, Fall M, Lancelot R, Diop A, Fall AG, Dicko A (2015). Modelling the abundances of two major *Culicoides* (Diptera: Ceratopogonidae) species in the Niayes area of Senegal. PLoS One.

[52] Akaike H. Information theory and an extension of the maximum likelihood principle. In: Second international symposium on information theory, Budapest: Akademinai Kiado: IEEE Int Symp Info; 1973. p. 267–81.

[53] R Core Team (2015). R: A language and environment for statistical computing.

[54] Dray S, Dufour AB (2007). The ade4 package: implementing the duality diagram for ecologists. J Stat Softw.

[55] Oksanen J, Blanchet FG, Kindt R, Legendre P, Minchin PR, O’Hara RB (2016). vegan: Community Ecology Package. R package version 2.3–5.

[56] Diallo M, Nabeth P, Ba K, Sall AA, Ba Y, Mondo M (2005). Mosquito vectors of the 1998–1999 outbreak of Rift Valley fever and other arboviruses (Bagaza, Sanar, Wesselsbron and West Nile) in Mauritania and Senegal. Med Ved Entomol.

[57] Ba Y, Diallo D, Dia I, Diallo M (2006). Comportement trophique des vecteurs du virus de la fièvre de la vallée du Rift au Sénégal: implications dans l’épidémiologie de la maladie. Bull Soc Pathol Exot.

[58] Talla C, Diallo D, Dia I, Ba Y, Ndione J-A, Morse A (2014). Statistical modeling of the abundance of vectors of West African Rift Valley fever in Barkédji, Senegal. PLoS One.

[59] Mondet B, Diaïté A, Fall AG, Chevalier V (2005). Relations entre la pluviométrie et le risque de transmission virale par les moustiques : cas du virus de la Rift Valley Fever (RVF) dans le ferlo (Sénégal). Environ Risque Sante.

[60] Gray JS, McIntyre A, Stirn J. Manuel des méthodes de recherche sur l’environnement aquatique: Evaluation biologique de la pollution marine, eu égard en particulier au benthos. Rome; Onzième partie, vol. 324: Food Agr Org; 1992.

[61] Simboura N, Zenetos A (2002). Benthic indicators to use in ecological quality classification of Mediterranean soft bottom marine ecosystems, including a new biotic index. Mediterr. Mar Sci.

[62] Jouan A, Coulibaly I, Adam F, Philippe B, Riou O, Leguenno B (1989). Analytical study of Rift Valley fever epidemic. Res Virol.

[63] Diallo M (1995). Dynamique comparée des populations de Culicidae à Kédougou (zone soudano-guinéenne) et à Barkédji (zone de savane sahélienne): conséquences dans la transmission des arbovirus.

[64] Mondet B, Diaïté A, Ndione JA, Fall AG, Chevalier V, Lancelot R (2005). Rainfall patterns and population dynamics of *Aedes* (*Aedimorphus*) *vexans* arabiensis, Patton 1905 (Diptera: Culicidae), a potential vector of Rift Valley fever virus in Senegal. J Vector Ecol.

[65] Janousek T, Kramer W (1999). Seasonal incidence and geographical variation of Nebraska mosquitoes, 1994–95. J Am Mosq Control Assoc.

[66] Reeves WC, Hardy JL, Reisen WK, Milby MM (1994). Potential effect of global warming on mosquito-borne arboviruses. J Med Entomol.

[67] Reiter P (2001). Climate change and mosquito-borne disease. Environ Health Persp.

[68] Ba Y, Diallo D, Kebe CMF, Dia I, Diallo M (2005). Aspects of bioecology of two Rift Valley fever virus vectors in Senegal (West Africa): *Aedes vexans* and *Culex poicilipes* (Diptera: Culicidae). J Med Entomol.

[69] Delatte H, Gimonneau G, Triboire A, Fontenille D (2009). Influence of temperature on immature development, survival, longevity, fecundity, and gonotrophic cycles of *Aedes albopictus*, vector of chikungunya and dengue in the Indian Ocean. J Med Entomol.

